# Decomposing socio-economic inequality in catastrophic out-of-pocket health expenditures in Malawi

**DOI:** 10.1371/journal.pgph.0000182

**Published:** 2022-02-08

**Authors:** Atupele N. Mulaga, Mphatso S. Kamndaya, Salule J. Masangwi

**Affiliations:** 1 Department of Mathematics and Statistics, Faculty of Applied Sciences, University of Malawi, Blantyre, Malawi; 2 School of Science and Technology, Malawi University of Business and Applied Sciences, Blantyre, Malawi; 3 Centre for Water, Sanitation, Health and Appropriate Technology Development (WASHTED), Malawi University of Business and Applied Sciences, Blantyre, Malawi; Egas Moniz Cooperativa de Ensino Superior CRL, PORTUGAL

## Abstract

Reducing health inequalities and inequities is one of the key goals that health systems aspire to achieve as it ensures improvement in health outcomes among all population groups. Addressing the factors contributing to inequality in catastrophic health expenditures is important to reducing inequality in the burden of health expenditures. However, there are limited studies to explain the factors contributing to inequalities in catastrophic health expenditures. The study aimed to measure and decompose socio-economic inequality in catastrophic health into its determinants. Data for the analysis come from the fourth integrated household survey. Data for 12447 households in Malawi were collected from April 2016 to April 2017 by the National Statistical Office. The secondary analysis was conducted from June 2021 to October 2021. Catastrophic health expenditure was estimated as a proportion of households whose out-of-pocket health expenditures as a ratio of non-food consumption expenditures exceeds 40% threshold level. We estimated the magnitude of socio-economic inequality using the Erreygers corrected concentration index and used decomposition analysis to assess the contribution of inequality in each determinant of catastrophic health expenditure to the overall socio-economic inequality. The magnitude of the Erreygers corrected concentration index of catastrophic health expenditure (CI = 0.004) is small and positive which indicates that inequality is concentrated among the better-off. Inequality in catastrophic health expenditure is largely due to inequalities in rural residency (127%), socio-economic status (-40%), household size (14%), presence of a child under five years old (10%) and region of the household (10%). The findings indicate that socio-economic inequality in catastrophic health expenditures is concentrated among the better-off in Malawi. The results imply that policies that aim to reduce inequalities in catastrophic health expenditures should simultaneously address urban-rural and income inequalities.

## Introduction

Health inequalities are systematic differences, variations and disparities in health outcomes among population groups [1]. One of the goals of health systems in both developed and developing countries is to reduce health inequalities in a way that improves the condition of the worse-off [2]. Socioeconomic inequalities in health are a great concern among policy makers as most of these inequalities are unjust and unfair and reflect inequality in the social determinants of health [1, 3, 4]. Studies globally have shown that determinants of health contributes greatly to inequality in health and health outcomes [3–8]. For example, a study in South Africa found that inequalities in social determinants of health such as social protection, employment, education and knowledge contributes greatly to inequalities in good self-assessed health [3]. This implies that policies that aim to reduce health inequalities should also be designed to address inequalities in social determinants of health.

Socio-economic inequality in out-of-pocket health expenditures may entail inequality in the burden of catastrophic health expenditures and worsen inequalities in access to and utilization of health services [2, 9, 10]. Prior studies that have assessed the magnitude of inequality in catastrophic health expenditures consistently reported that catastrophic expenditure is concentrated among the worse-off [11–17]. For instance, studies have reported that catastrophic health expenditure is concentrated among the worse-off households and that socioeconomic status, household size, having elderly household members greatly contribute to inequality in catastrophic expenditures [15, 16]. Similar studies in Sub-Saharan African countries have reported that catastrophic health expenditure is concentrated among the worse-off households [11–13]. However, there is limited evidence on decomposing inequality in catastrophic expenditures to understand how inequality in the determinants contribute to inequality in catastrophic health expenditures in sub-Saharan Africa countries. We extend the studies conducted in Sub-Saharan Africa countries by decomposing socio-economic inequality in catastrophic health expenditures in Malawi.

In Malawi the incidence of catastrophic health expenditures was estimated at 0.73% at 40% threshold level of nonfood expenditures in 2011 and using similar data another study estimated the incidence of catastrophic health expenditures at 1.37% at the same threshold level in 2016 [18, 19]. These results indicated an increase in the incidence of catastrophic health expenditures over the five-year period. Nevertheless, the studies did not report socio-economic inequality in catastrophic health expenditures and how inequality in the determinants of catastrophic expenditures contributes to the overall socio-economic inequality.

Analysis of inequalities in Malawi has shown that health inequalities are interrelated to wealth, education, regional and gender inequalities [20]. These inequalities reinforce one another and may require policies that simultaneously address such inequalities [20]. For example, utilization of maternal health services is low among women with lower education, residing in rural areas and in lower wealth quintile in Malawi [21]. These inequalities contribute to poor maternal health to the disadvantage of the worse-off. This is also the case with out-of-pocket health expenditures which is concentrated among the better-off [22]. This study [22] also reported that income and education inequalities contributed to the majority of inequalities in out-of-pocket health expenditures. Such inequalities in health expenditures may exacerbate inequalities in access and utilization consequently inequality in catastrophic health expenditures. However, to the best of our knowledge there is no study that has assessed and decomposed inequality in catastrophic health expenditure in Malawi. Therefore, the aim of the study was to assess and decompose inequality in catastrophic health expenditures into its determinants. The study adds to the existing literature on health inequalities by providing evidence on the major determinants that contribute to inequality in catastrophic health expenditures in a Sub Saharan Africa country. This will help policy makers to understand the magnitude of inequality, the factors contributing to inequality in catastrophic health expenditures and design policies to simultaneously address inequality in catastrophic expenditures and its determinants.

## Methods

### Study design

The study uses a cross-sectional design using secondary data from a nationally representative survey conducted from April 2016 to April 2017.

### Data source and definition of variables

Data for the study come from the fourth integrated household survey (IHS4). Data were collected from April 2016 to April 2017 by the National Statistical Office of Malawi. This secondary reanalysis of the data was conducted between June 2021 to October 2021. The Malawi fourth integrated household is a cross sectional survey that uses a two stage sampling design to select the households. The first stage involved selecting 780 enumeration areas which were stratified by urban and rural strata and were selected with probability proportional to size and the second stage involved selecting 16 primary households and 5 replacement households from the sampling frame of households in each sampled enumeration area using random systematic sampling. The paper used data for a total sample of 12,447 households which included 53,885 individuals. Data collected include information on household characteristics and demographics on each household member, education, food and nonfood consumption expenditures and health.

### Outcome variable and covariates

The outcome variable is dichotomous taking the value 1 if a household faced catastrophic health expenditure and 0 otherwise. A household faced catastrophic health expenditure if out-of-pocket health expenditure as a proportion of household capacity to pay exceed 40% threshold level where capacity to pay was defined as household total annual consumption expenditures minus food expenditures.

The covariates included age of household head, sex of household head, household socioeconomic status based on household consumption expenditure per capita and categorized into five quintile groups from poorest to richest, having at least one child under five year old in household or not, having an elderly member in household or not, having at least one hospitalized member in the past year or not, rural or urban household location, region, type of health facility with medical doctor, household size, distance to the nearest health facility.

### Ethical clearance

We obtained ethical clearance for the secondary analysis from the National Committee on Research in the Social Sciences and Humanities (NCRSH) reference No. P.10/19/434.

### Statistical analysis

#### Measuring inequality in catastrophic health expenditures

We estimated inequality in catastrophic health expenditures using the concentration index. The concentration index is a common measure used in the literature to assess income related inequality in health variables. The concentration index measures the degree in socioeconomic inequality of a health variable of interest and is defined as two times the area between the line of inequality and the concentration curve [23]. The concentration curve plots the cumulative proportion of the health variable on the y-axis against the cumulative proportion of the sample ranked by socioeconomic status from the poorest to the wealthiest on the x-axis [4]. The index lies between -1 and +1 when the health variable of interest is unbounded. However, for bounded health variables Wagstaff [24] has shown that the concentration index lies between *μ*−1 and 1−*μ* for large samples. Positive values of the concentration index indicate that inequality is more concentrated among the better-off and negative values indicate that inequality in more concentrated among the worse-off [25]. The concentration index was estimated using the convenient covariance formula as [25]:

$$C=\frac{2}{\mu }cov(y_{i},r_{i})$$ (1)

Where *r*_*i*_ is the fractional rank of *i*^*th*^ household across socioeconomic status as measured by consumption expenditure per capita in this study, *y*_*i*_ is the health variable of interest which is the incidence of catastrophic expenditures and *μ* is the mean of *y*_*i*_.

For a dichotomous health variable of interest, Wagstaff [24] proposed a normalized concentration index obtained by dividing the standard concentration index in Eq (1) by either the reciprocal of *y*_*i*_ or the upper bound of the concentration index of *y*_*i*_. However, Erreygers [26] has shown that rank dependent measures of socioeconomic inequality such as the Wagstaff concentration index should satisfy four properties. These include; (i) the mirror image property which states that for any given health distribution the index of a health variable is equal in absolute value to the index of ill-health variable with opposite sign, (ii)cardinal invariance property which states that a positive linear transformation of the health variable does not change the value of index, (iii) transfer property which states that any mean preserving change in health distribution in favor of the wealthier result in change in index in favor of the wealthier and this is also true for change in health distribution in favor of the worse-off, (iv) level of independence property which states that the value of the index does not change with change in health levels of all persons by an equal absolute amount. Whereas the Wagstaff concentration index satisfy properties (i) to (iii) it fails to satisfy the level of independence property. Thus, for bounded health variables, Erreygers [26] proposed a corrected concentration index which satisfies all the properties of rank dependent measures of inequality. In this paper we computed the Erreygers corrected concentration index since our outcome variable is a bounded dichotomous variable. The Erreygers corrected concentration index was estimated as follows [26]:

$$EI=\frac{4\mu }{y^{max}-y^{min}}CI$$ (2)

Where *μ* is the mean of catastrophic health expenditures, *y*^*max*^ *and y*^*min*^ are the upper bound and lower bound of catastrophic health expenditures respectively and *CI* is the concentration index of catastrophic health expenditures which was obtained using (1). The paper used conindex command in Stata 15 [27] to compute the concentration indices. Stata 15 was also used to decompose the concentration index of catastrophic expenditures into its determinants.

#### Decomposing socio-economic inequality in catastrophic health expenditures into its determinants

The paper used a decomposition analysis to assess the contribution of inequality in each determinant of catastrophic health expenditures to the overall socioeconomic inequality. The method proposed by Wagstaff et al. [4] is used to decompose socioeconomic inequality in catastrophic health expenditures into its determinants. This method has also been used by other authors to decompose inequality in catastrophic health expenditures [15, 16, 28, 29]. Decomposing the concentration index allows us to understand how inequality in each determinant of catastrophic health expenditure contributes to overall socioeconomic inequality in catastrophic health expenditures. This is important for policy makers to design interventions to tackle inequality in the determinants and consequently inequality in catastrophic health expenditures. The method of decomposing the concentration index as proposed by Wagstaff et al. [4] is based on the linear regression model that relates a continuous health outcome variable *y*_*i*_ to a set of k determinants *x*_*k*_, given as follows:

$$y_{i}=\alpha +\sum_{k}\beta_{k}x_{ki}+\varepsilon_{i}$$ (3)

Where *β*_*k*_ is the vector of regression coefficients, *x*_*k*_ is a set of k determinants and *ε*_*i*_ is the random error term. Wagstaff et al. [4] has shown that the concentration index of *y*, denoted by *C*_*y*_ can be decomposed as follows:

$$C_{y}=\sum_{k}\left(\frac{\beta_{k}{\bar{x}}_{k}}{\mu }\right)C_{k}+\frac{{GC}_{\varepsilon }}{\mu }$$ (4)

Where *μ* is the mean for the outcome variable *y*, ${\bar{x}}_{k}$ is the mean of each determinant, *C*_*k*_ is the concentration index for the determinants, *β*_*k*_ represents the estimated regression coefficients for each determinant factor obtained from Eq (3) and *GC*_*ε*_ is the generalized concentration index for the error term. For the Erreygers corrected concentration index a similar decomposition formula for the index is expressed as follows [26]:

$$EI=4(\sum_{k}\beta_{k}({\bar{x}}_{k}C_{k})+{GC}_{\varepsilon })$$ (5)

Where ${\bar{x}}_{k}$ is the mean of each determinant in the regression analysis, *C*_*k*_ is the concentration index for the determinants and *β*_*k*_ is the estimated regression coefficient or marginal effect.

The concentration index *C*_*y*_ and *EI* for the outcome variable in (4) and (5) respectively is decomposed into two components. The first component represents the explained inequality due to variation in the explanatory variables across socioeconomic status and the second component represents inequality that cannot be explained by variation in the explanatory variables across socioeconomic status [4, 5, 25].

For the decomposition analysis in this paper we used multilevel logistic regression model since our outcome variable is dichotomous taking the value 1 if a household faced catastrophic health expenditure and zero otherwise. In addition, the survey data used is hierarchically structured where households are nested in sub districts hence the multilevel logistic regression account for the hierarchical structure of the data to give correct inference on the estimated parameters of the regression model.

To decompose the overall socioeconomic inequality in catastrophic health expenditures, we first estimated a multilevel logistic regression to obtain the marginal effects indicating the intensity of the relationship between catastrophic expenditures and its determinants. The marginal effects were used together with the estimated concentration indices of each determinant indicating inequality in each determinant and the estimated mean of each determinant in computing the contribution of each determinant to the overall socio-economic inequality in catastrophic health expenditures using Eq (5). The contribution of each determinant to overall inequality was obtained as four times the product of the marginal effect, the estimated concentration index and estimated mean of each determinant. A positive contribution by a variable indicates that the variable increases inequality in catastrophic health expenditures disfavoring the worse-off and a negative contribution indicates reduction in inequality [4, 16].

The decomposition analysis proposed by Wagstaff et al. [4] requires that the regression model relating the health outcome variable such as catastrophic health expenditures to a set of k determinants *x*_*k*_ to be linear in form. However, the logistic regression model used in this paper is nonlinear in form. To deal with this problem we used the logit linear transformation of the logistic regression model as proposed by other authors [5, 30]. This enables the decomposition of the concentration index to be implemented in the same way as proposed by Wagstaff et al. [4] in Eq (4). We used the logit linear transformation on the logistic regression model and the marginal effects of the regression coefficients in the decomposition analysis. Other authors have also used linear transformation of the nonlinear models in decomposing inequality in catastrophic health expenditures [15, 16, 28, 31].

The multilevel logit linear transformation model used in the decomposition analysis is specified as follows:

$$\ln\left(\frac{\pi_{ij}}{1-\pi_{ij}}\right)=\alpha_{ij}+\sum \beta_{i}^{m}x_{ij}+u_{j}$$ (6)

Where *π*_*ij*_ is the probability of incurring catastrophic health expenditure, $\beta_{i}^{m}$ represents a vector of the estimated regression marginal effects of the corresponding determinant factors *x*_*ij*_ and *u*_*j*_ is the higher level random error term. Analysis was implemented using Stata 15 and we adjusted for sampling design using survey sample weights and the survey set command. Results were interpreted at 5% significance level.

## Results

Table 1 shows the summary statistics of catastrophic health expenditure and its determinants. More than 71% of the households were male headed and over 26% of the household heads were aged 26 to 35 years old. More than half (53.5%) of the households had at least one child under five years’ old. On average households had four members. Only 20% of the households had an elderly household member, 22% had at least one household member chronically ill and 13% had at least one household member hospitalized in the 30 days preceding the survey. Majority (81%) of the sampled households were rural. On average the distance to nearest health facility was 13 km and about 87% of the households reported government health facility as the nearest facility where medical doctors were based. On average the total annual household consumption expenditure was MWK 831433 and the household total annual out-of-pocket health expenditures was MWK 15649. Only 1.3% of the sampled households faced catastrophic health expenditures at 40% level of non-food expenditures. About 3%, 6% and 14% faced catastrophic health expenditures at 30%,20% and 10% threshold level respectively (results not reported in Table 1).

**Table 1:** Summary statistics of sampled households (n = 12447).

Variable	Weighted Mean(SD)/percentage
Catastrophic health expenditure Age of household head	1.34
Less than 26 years	12.30
26–35 years	26.66
36–45 years	23.79
46–55 years	15.21
Over 56 years	22.04
Male headed household	71.12
Size of household	4.29(2.00)
Have at least one child under 5 years	53.52
Have at least one elderly member greater than 60 years	19.75
Have at least one chronically ill member	22.33
Have at least one hospitalized member	13.16
Rural location	80.95
Distance to the nearest health facility (KM)	13.33(16.85)
Type of health facility	
Government	87.23
Religious	10.68
Private	2.08
Region	
Northern	9.15
Central	44.32
Southern	46.53
Total annual consumption expenditure (MWK)	831433(94289)
Total annual health expenditure (MWK)	15649(7449853)

### Socioeconomic inequality in catastrophic health expenditure and decomposition analysis

Table 2 reports the estimated socio-economic inequality in catastrophic health expenditures and each of the covariate associated with catastrophic health expenditures as measured by the concentration index. The concentration Index(CI) of incurring catastrophic health expenditure (*CI* = 0.004, *p*<0.10) indicates that inequality in catastrophic health expenditure is moderate and concentrated among better-off households. Female headed household (*CI* = −0.086, *p*<0.01), presence of at least one child under five years in the household (*CI* = −0.282, *p*<0.01), larger household size with six to eleven members (*CI* = −0.251, *p*<0.01), residency in rural areas (*CI* = −0.363, *p*<0.01), longer distance to the nearest health facility (*CI* = −0.064, *p*<0.01) and access to religious health facility with medical doctor (*CI* = −0.032, *p*<0.01) is concentrated amongst poor households. On the other hand, having at least one household member hospitalized (*CI* = 0.018, *p*<0.01) and access to private health facility with medical doctor (*CI* = 0.014, *p*<0.01) is concentrated amongst rich households.

**Table 2:** Erreygers corrected concentration indices for catastrophic health expenditures and its determinants.

Variable	Concentration index (Std.Error)	P-value
Catastrophic health expenditure (CHE)	0.004(0.0024)	0.099*
Age of household head(ref = ≥ 56 years)		
Less than 26 years	0.029(0.007)	0.001***
26–35 years	0.029(0.009)	0.0013**
36–45 years	-0.054(0.009)	0.001***
46–55 years	-0.010(0.007)	0.177
Female household head	-0.086(0.009)	0.000***
Size of household (ref ≤ **5** members)		
6–11 members	-0.2514(0.009)	0.001***
≥ 12 members	-0.0036(0.001)	0.001***
Socio-economic status		
Quintile 2	-0.311(0.008)	0.001***
Quintile 3	0.001(0.008)	0.991
Quintile 4	0.320(0.008)	0.001***
Quintile 5(Richest)	0.639(0.006)	0.001***
Have at least one child	-0.282(0.010)	0.001***
Have at least one elderly member	-0.005(0.008)	0.535
Have at least one chronically ill member	-0.009(0.009)	0.267
Have at least one hospitalized member	0.018(0.007)	0.009**
Rural location	-0.363(0.012)	0.001***
Distance to the nearest health facility (ref = ≤ **34** Km)		
35–69 Km	-0.064(0.006)	0.001***
≥ **70** Km	-0.013(0.003)	0.001***
Type of health facility (ref = government)		
Religious	-0.032(0.006)	0.001***
Private	0.014(0.003)	0.001***
Region (ref = Northern)		
Central	0.081(0.010)	0.001***
Southern	-0.094(0.010)	0.001***

*p<0.10

**p<0.05

***p<0.01

Table 3, gives results on decomposing socio-economic inequality in catastrophic health expenditure into its determinants. The analysis was conducted to assess the contribution of inequality in each determinant of catastrophic health expenditures to the overall socio-economic inequality in catastrophic health expenditures. Column two gives the marginal effect estimated from the fitted regression model. The column indicates the magnitude of the relationship between each determinant and catastrophic health expenditure after controlling for all other determinants. For example, the predicted probability of catastrophic health expenditures was 0.028 greater for households with hospitalized members. The probability of facing catastrophic expenditure was 0.01 greater for rural household and 0.013 greater for households located in central regions. For households with a larger family from 6 to 11 members the probability of facing catastrophic health expenditures was 0.01 greater and it was also 0.01greater for households accessing health services at religious health facilities than government facilities. Compared with households in lower income quintile the probability of facing catastrophic health expenditures was 0.01greater in the richest income quintile.

**Table 3:** Decomposition analysis of concentration index for catastrophic health expenditures.

Independent variables	Marginal effects	Weighted Mean	*C*_*k*_	Contribution to *C*_*y*_	Contribution to *C*_*y*_ (%)
Age of household head (ref = ≥ 56 years)					-1
≤26 years	-0.0091	0.123	0.0294	-0.0001	
26–35 years	-0.0053	0.267	0.0295	-0.0001	
36–45 years	-0.0067	0.238	-0.0541	0.0003	
46–55 years	-0.0078	0.152	-0.0100	0.00005	
Female household head	0.0009	0.289	-0.0857	-0.0001	1
Household size (ref ≤ **5** members)					14
6–11 members	0.0066*	0.256	-0.2514	-0.002	
≥ 12 members	0.0152	0.0196	-0.0036	-0.0000003	
Socio-economic status (ref = Quintile1)					-40
Quintile 2	0.0071*	0.2	-0.3199	-0.0019	
Quintile 3	0.0093*	0.199	0.0009	0.0000007	
Quintile 4	0.0093*	0.2	0.32032	0.00247	
Quintile 5(Richest)	0.0097*	0.199	0.6397	0.005235	
Have at least one child	0.0025	0.535	-0.2825	-0.00141	10
Have at least one elderly member	-0.0034	0.198	-0.0051	0.00001	-0.1
Have at least one chronically ill member	0.0035	0.223	-0.0096	-0.00003	0.21
Have at least one hospitalized member	0.0178*	0.132	0.0182	0.00018	-1.24
Rural location	0.0147*	0.809	-0.3630	-0.018538	127
Distance to health facility (ref = ≤ **34** Km)					0.14
35–69 Km	-0.0012	0.098	-0.0644	-0.000028	
≥70 Km	-0.0067	0.0196	-0.0133	0.0000077	
Type of health facility (ref = government)					0.80
Religious	0.0082*	0.107	-0.0316	-0.00011	
Private	-0.0063	0.021	0.0143	-0.0000069	
Region (ref = Northern)					-10
Central	0.0122*	0.443	0.0798	0.00179142	
Southern	0.0008	0.465	-0.0944	-0.00028	

*significant at 5% level.

Column three gives the weighted mean for each of the determinants associated with catastrophic health expenditures and column four gives the estimated concentration index for each of the determinants.

The contribution of socio-economic inequality in each determinant to the overall socio-economic inequality is estimated in column five. This column of the absolute contribution is estimated by multiplying four to the product of marginal effects, weighted mean and the Erreygers corrected concentration index of the determinant as described in Eq (5). For example, the absolute contribution of residency in rural areas is estimated by 4[0.0097*0.809*(-0.3630)] and the relative contribution was obtained by dividing the absolute contribution by the total contribution of all the determinants. As shown by the relative contributions in the last column of Table 3; the majority of socioeconomic inequality in catastrophic expenditure was mainly due to inequality in residency in rural areas (127%), household socio-economic status (-40%), household size (14%), region in which a household is located (-10%) and having children under five years (10%). Other determinants of catastrophic health expenditure such as female headed household, having at least one elder member in the household, having at least one hospitalized member, having of one chronically ill member, access to nearest health facility with medical doctor and distance to the nearest health facility contributed marginally to inequality in catastrophic health expenditure. In total, inequalities in these determinants accounted for only 2% of the total inequality in catastrophic health expenditures.

## Discussion

This study aimed at measuring and decomposing socioeconomic inequality in catastrophic health expenditures to assess the contribution of inequality in each determinant of catastrophic health expenditures to the overall inequality. The findings show that socioeconomic inequality is marginally significant and concentrated among the better-off households. Majority of the socioeconomic inequality in catastrophic health expenditures is due to inequalities in residency in rural area, socioeconomic status, household size, having at least a child under five years old and region in which household is located. We discuss these findings in the paragraphs that follows.

Firstly, contrary to findings from previous studies [11, 13–16, 32–34] the results demonstrate that catastrophic health expenditure is concentrated among better-off households in Malawi. This could be attributed to the challenges faced by free public health services delivery in Malawi such as constant stock out of drugs, poor quality of services, shortage of human resources which forces the better-off to seek high quality care in private facilities putting households at risk of incurring catastrophic expenditure [20, 35, 36]. This is also supported by our finding in Table 2 which indicates that access to private health facility is more concentrated among the better-off. Furthermore, other studies have shown that the use of health care services and out-of-pocket health expenditures are more concentrated among the better-off households in Malawi [22, 37]. This high out-of-pocket health expenditures among the better-off increase the likelihood of incurring catastrophic health expenditures.

Another plausible explanation is that due to their ability to pay the better-off households use private health care more than the worse-off as such they incur high out-of-pocket health expenditures putting them at risk of catastrophic health expenditures. A health system that gives access to high quality care to the rich due to their ability to pay leaving lower quality care to the poor is inequitable and against the core values of universal health coverage goal [38]. Malawi has a long history of providing free public health services to reduce inequality and inequity in health services utilization and financial protection however it has been observed that inequities in access and health services utilization still persists [39] this exacerbates inequalities in health expenditures [22] consequently inequalities in catastrophic health expenditures between the worse-off and better-off. This finding reinforces the need to improve the health systems challenges such as poor quality of care, shortages of drugs and human resources to reduce inequalities in use and access consequently inequalities in health expenditures.

Secondly, our findings that socioeconomic status, residency in rural areas and household size are the major contributors to socioeconomic inequality in catastrophic expenditure are consistent with findings from previous studies [15, 16]. However, we find that socioeconomic status contributes negatively to inequality in catastrophic health expenditure which indicates that socioeconomic status decreases inequality in catastrophic health expenditure. This shows that the combined effect of the marginal effect of socio-economic status on catastrophic health expenditures and its inequality is to reduce inequality in catastrophic health expenditures such that catastrophic health expenditures is greater among the better-off. There are huge income inequalities in Malawi such that these income inequalities and other health inequalities are interrelated [20]. For example, a study in Malawi found that inequality in out-of-pocket expenditures is more concentrated among the rich and the majority of these inequalities are influenced by income inequality [22]. Thus, in the case of Malawi increasing household socioeconomic status has an effect of decreasing inequality in catastrophic health expenditure. Policies that aim to address inequality in catastrophic out-of-pocket health expenditures should also address income and other related inequalities. This could be through social cash transfer interventions to poor households which could help to reduce income inequalities.

Thirdly, we find that residency in rural areas contributes to the majority of socioeconomic inequality in catastrophic health expenditures. The relative positive contribution to socioeconomic inequality indicates that residency in rural areas increases inequality in catastrophic expenditure disfavoring the poor. Huge rural–urban income inequalities coupled with poor geographic accessibility of public health facilities in rural areas creates inequality in access to and use of health services disfavoring poor rural households in Malawi [20]. Due to poor geographical accessibility of public facilities poor rural households may incur other costs associated with seeking care such as transportation which puts them at risk of catastrophic health expenditures as observed by other studies in Kenya and Zambia [32, 34]. In Malawi, about 40% of health services in rural areas are provided by Christian Health Association of Malawi(CHAM) health facilities which charge user fee [20, 40] as such even smallest expenditures by poor households seeking care at religious health facilities can drive them into catastrophic health expenditures. Moreover, our analysis show that access to such mission/religious health facilities is concentrated among poor households which means rural poor households disproportionately use religious health facilities more creating inequality in health expenditures disfavoring poor households. The government of Malawi introduced service level agreements (SLAs) with Christian Health Association of Malawi (CHAM) service providers in 2005 which allow poor rural households to access free health care in these mission facilities without facing financial hardship [41]. However, our finding that residency in rural areas contributes to inequality in catastrophic health expenditure disfavoring poor households imply that the SLAs may not have achieved its intended purpose of protecting households and reducing health expenditure disparities in rural areas. Nevertheless these SLAs have a potential to improve financial protection from the risk of illnesses among vulnerable population groups as observed by a previous study [42]. It is possible that many of the rural CHAM facilities and essential services are not included in the SLAs and poor households who access care in these health facilities face catastrophic health expenditure increasing inequality disfavoring the poor in rural households. The plans by government to improve the SLAs to include more health facilities and essential services should be pursued. This coupled with improving quality of services and geographic accessibility of public health facilities in rural areas could help to reduce the inequality in access and consequently reduce inequality in catastrophic expenditures.

The study has limitations. The study uses cross sectional data which prevents causal interpretation of the relationship between catastrophic health expenditures and its determinants used in the decomposition analysis. The use of self-reported data on household consumption expenditures may introduce recall bias which can lead to underestimation or overestimation of catastrophic health expenditures. The analytical method for estimating catastrophic health expenditures does not count households that forgo care due to inability to pay. In addition, households that borrow to finance health care may increase their consumption expenditures and may be classified into higher expenditure quintiles. These limitations may underestimate or overestimate the incidence of catastrophic health expenditures.

## Conclusion

The findings of the study have shown that socioeconomic inequality in catastrophic expenditures is more concentrated among better-off households. Majority of the inequality in catastrophic health expenditures is due to inequality in residency in rural areas, socioeconomic status, region in which the household is located, household size and having children under five years. The findings suggest that government policies and programs that aim to reduce inequality in catastrophic health expenditure should simultaneously reduce income, rural-urban and regional related inequalities. A future study should explore whether low catastrophic health expenditures among the worse off in Malawi is a result of households experiencing financial protection or is simply as a result of forgoing health care to avoid catastrophic health expenditures.
